# Supplementary material for: Identification of the Centrifugation-Caused Paralytic Impact on Neutrophils
Source: Cells. 2025 Aug 30;14(17):1350. doi: 10.3390/cells14171350 (PMC12427856; doi:10.3390/cells14171350)
Supplement: Supplementary file 1 [file cells-14-01350-s001.zip › cells-3836694-supplementary.pdf]

Table S1: Median AFU values with interquartile ranges for surface antigen expression under different conditions

Incubation time	Isolation method	CD11b total median [AFU] (IQR)	CD11b activated median [AFU] (IQR)	Ratio CD11b total/ CD11b activated median [AFU] (IQR)	CD62L median [AFU] (IQR)	CD66b median [AFU] (IQR)
0h	1<br>(DGC at 756g for 30 min)	1175<br>(658)	3105<br>(1593)	2,5<br>(2,1)	13889<br>(5538)	6177<br>(2327)
	2<br>(sedimentation at 1g for 60 min)	1713<br>(984)	2965<br>(1565)	1,9<br>(1,0)	12502<br>(5082)	7304<br>(2255)
	3<br>sedimentation at 1g for 60 min, overlay with plasma, followed by DGC at 756g for 30 min	1438<br>(589)	2989<br>(2309)	1,8<br>(1,3)	14246<br>(6400)	7555<br>(2017)
	4<br>sedimentation at 1g for 60 min, overlay with erythrocytes, followed by DGC at 756g for 30 min	1113<br>(726)	2455<br>(1174)	2,4<br>(1,9)	14101<br>(5940)	6111<br>(3067)
2h	1<br>(DGC at 756g for 30 min)	1883<br>(1631)	2881<br>(1024)	1,3<br>(0,6)	8466<br>(6884)	8569<br>(5354)
	2<br>(sedimentation at 1g for 60 min)	6975<br>(1897)	19475<br>(9133)	2,8<br>(1,1)	2366<br>(4301)	17944<br>(10154)
	3<br>sedimentation at 1g for 60 min, overlay with plasma, followed by DGC at 756g for 30 min	2249<br>(881)	2652<br>(1276)	1,3<br>(0,7)	4129<br>(4096)	9696<br>(5355)
	4<br>sedimentation at 1g for 60 min, overlay with erythrocytes, followed by DGC at 756g for 30 min	2101<br>(1399)	3175<br>(1672)	1,2<br>(0,6)	10241<br>(6310)	8290<br>(5009)
22h	1<br>(DGC at 756g for 30 min)	2546<br>(1496)	2819<br>(1074)	1,0<br>(0,5)	290<br>(243)	11136<br>(4366)
	2<br>(sedimentation at 1g for 60 min)	6536<br>(3334)	11358<br>(6573)	2,0<br>(0,9)	500<br>(280)	19249<br>(17851)
	3<br>sedimentation at 1g for 60 min, overlay with plasma, followed by DGC at 756g for 30 min	3091<br>(1409)	3517<br>(864)	1,3<br>(0,7)	309<br>(178)	14548<br>(6123)
	4<br>sedimentation at 1g for 60 min, overlay with erythrocytes, followed by DGC at 756g for 30 min	2824<br>(858)	3103<br>(2004)	1,2<br>(0,6)	283<br>(276)	12093<br>(7589)
